# 2891. Nebulized Phage Therapy for Patients with Cystic Fibrosis with Chronic *Pseudomonas aeruginosa* Pulmonary Infection: A Phase 1b/2a Randomized, Double-Blind, Placebo-Controlled, Multicenter Study

**DOI:** 10.1093/ofid/ofad500.2474

**Published:** 2023-11-27

**Authors:** Urania Rappo, Ariel Cohen, Edith Kario, Jenia Gold, Hadas Tamar Nevenzal, Inbal Levy-Saar, Tal Cohen, Iddo Weiner, Hila Sberro Livnat, Ilya Vainberg Slutskin, Jagoda Jablonska, Tim Axelrod, Ori Bahar, Nufar Buchshtab, Vered Lev, Yaron Tzur, Yulia Zarchin, Myriam Golembo, Regis Vilchez, Eitan Kerem, Merav Bassan

**Affiliations:** BiomX, Branford, CT; BiomX, Ltd, Ness Ziona, HaMerkaz, Israel; BiomX, Ltd, Ness Ziona, HaMerkaz, Israel; BiomX, Ltd, Ness Ziona, HaMerkaz, Israel; BiomX, Ltd, Ness Ziona, HaMerkaz, Israel; BiomX, Ltd, Ness Ziona, HaMerkaz, Israel; BiomX, Ltd, Ness Ziona, HaMerkaz, Israel; IW, Ness Zionna, HaMerkaz, Israel; BiomX, Ltd, Ness Ziona, HaMerkaz, Israel; BiomX, Ltd, Ness Ziona, HaMerkaz, Israel; BiomX, Ltd, Ness Ziona, HaMerkaz, Israel; BiomX, Ltd, Ness Ziona, HaMerkaz, Israel; BiomX, Ltd, Ness Ziona, HaMerkaz, Israel; BiomX, Ltd, Ness Ziona, HaMerkaz, Israel; BiomX, Ltd, Ness Ziona, HaMerkaz, Israel; BiomX, Ltd, Ness Ziona, HaMerkaz, Israel; BiomX, Ltd, Ness Ziona, HaMerkaz, Israel; BiomX, Ltd, Ness Ziona, HaMerkaz, Israel; BiomX, Inc, Cambridge, Massachusetts; Hadassah University Hospital, Jerusalem, Yerushalayim, Israel; MB, Ness Zionna, HaMerkaz, Israel

## Abstract

**Background:**

*Pseudomonas aeruginosa* (PsA) continues to cause difficult-to-treat pulmonary infections and hospitalizations in patients with cystic fibrosis (CF). Safer and targeted therapies are needed to minimize the morbidity and disease burden of drug resistant PsA isolates.

**Methods:**

This Phase 1b/2a randomized, double-blind, placebo-controlled, multicenter study is evaluating the safety and tolerability of nebulized phage (BX004-A) and its effect on sputum *PsA* burden and clinical outcomes. Phage therapy is administered on top of standard of care inhaled antibiotics (tobramycin, aztreonam, or colistin) in at least 32 adult CF subjects with clinically stable lung disease (FEV1 at least 40%) and chronic PsA pulmonary infection. Subjects are randomized if all sputum PsA isolates from Screening are susceptible to at least 1 phage in BX004-A. In Part 1 (single-ascending and multiple dose portion), subjects were randomized (3:1) to BX004-A or placebo x7d, plus their usual inhaled antibiotic (D1-7). In Part 2 (multiple dose portion), subjects are randomized (2:1) to twice daily BX004-A or placebo x10d, plus their usual inhaled antibiotic (D1-28).

**Results:**

In Part 1, 9 subjects were randomized (7 on BX004-A, 2 on placebo), with a mean baseline PsA burden of 7.4 (range 4.2-8.5) and 7.9 (range 7.8-8.0) log_10_ colony forming unit (CFU)/g in BX004-A vs placebo, respectively. One subject in each arm had a multi-drug resistant PsA and 1 subject in each arm had an extensively drug-resistant PsA. Mean PsA CFU reduction at D15 (compared to baseline) was -1.42 log (BX004-A) vs. -0.28 log (placebo). BX004-A was well-tolerated with no treatment-related adverse events. Each subject was consistently colonized with the same genotypic strain of PsA by next-generation sequencing, from Screening up to end of therapy, with no emerging phage resistance in treated subjects. Phage was detected in the sputum of all BX004-A subjects during treatment, including in some subjects up to D15. Part 2 is ongoing with comparable demographics and baseline characteristics.

**Conclusion:**

Part 1 showed that BX004-A was well-tolerated with notable microbiologic efficacy. All Part 1 subjects had high levels of Screening *PsA* with all morphotypes susceptible to the phage cocktail.

**Disclosures:**

**Urania Rappo, MD**, BiomX: employee and may own stock **Ariel Cohen, PhD**, BiomX: employee and may own stock **Edith Kario, PhD**, BIomX: employee and may own stock **Jenia Gold, M.Sc**, BiomX: employee and may own stock **Hadas Tamar Nevenzal, PhD**, BiomX: employee and may own stock **Inbal Levy-Saar, M.Sc**, BiomX: employee and may own stock **Tal Cohen, M.Sc**, BiomX: employee and may own stock **Iddo Weiner, PhD**, BiomX: employee and may own stock **Hila Sberro Livnat, PhD**, BiomX: employee and may own stock **Ilya Vainberg Slutskin, PhD**, BiomX: employee and may own stock **Jagoda Jablonska, PhD**, BiomX: employee and may own stock **Tim Axelrod, PhD**, BiomX: employee and may own stock **Ori Bahar, B.Sc**, BiomX: employee and may own stock **Nufar Buchshtab, M.Sc**, BiomX: employee and may own stock **Vered Lev, PhD**, BiomX: employee and may own stock **Yaron Tzur, M.Sc**, BiomX: employee and may own stock **Yulia Zarchin, M.Sc**, BiomX: employee and may own stock **Myriam Golembo, PhD**, BiomX: employee and may own stock **Regis Vilchez, MD, PhD**, BiomX: Advisor/Consultant|BiomX: may own stock **Eitan Kerem, MD**, BiomX: Advisor/Consultant **Merav Bassan, PhD**, BiomX: employee and may own stock

